# Relationship of Ferritin and Procalcitonin with SOFA-2 Scores in Intensive Care Patients with COVID-19-Associated Sepsis: A Cross-Sectional Analysis

**DOI:** 10.3390/biomedicines14071413

**Published:** 2026-06-23

**Authors:** Murat Ay, Semiha Orhan, Nese Demirtürk, Erhan Bozkurt, Alper Sari, Merve Ay

**Affiliations:** 1 Department of Internal Medicine, Kütahya State Hospital, Kütahya 43100, Turkey; 2 Department of Intensive Care Unit, Afyonkarahisar Health Sciences University, Afyonkarahisar 03030, Turkey; smhorhan@gmail.com; 3 Department of Infectious Diseases and Clinical Microbiology, Afyonkarahisar Health Sciences University, Afyonkarahisar 03030, Turkey; nesedemirtuk@yahoo.com; 4 Department of Internal Medicine, Afyonkarahisar Health Sciences University, Afyonkarahisar 03030, Turkey; drerhanbozkurt@gmail.com (E.B.); alpersari_@hotmail.com (A.S.); 5 Department of Anesthesiology and Reanimation, Kütahya Evliya Çelebi Training and Research Hospital, Kütahya 43100, Turkey; mrvkcsln.dr@gmail.com

**Keywords:** COVID-19, sepsis, ferritin, procalcitonin, SOFA score

## Abstract

**Background/Objectives**: We investigated the association of serum ferritin and procalcitonin (PCT) with Sepsis-related Organ Failure Assessment (SOFA)-2 score-based organ dysfunction severity in intensive care patients with COVID-19-associated sepsis. **Methods**: Patients were stratified by day 5 ferritin (ng/mL) and PCT (μg/L) levels; associations were analysed across severity groups defined by an SOFA-2 score of <5 (mild) or ≥5 (severe). **Results**: Day 5 PCT did not predict the SOFA-2 score (*p* > 0.05). The optimal day 5 ferritin cut-off was >1191 ng/mL (35.78% sensitivity, 82.38% specificity; area under the curve (AUC) = 0.608). Day 5 ferritin was associated with SOFA-2 severity in the univariable analysis but did not remain an independent correlate after adjustment for C-reactive protein (CRP) and lactate dehydrogenase (LDH); in a mortality model, neither ferritin nor PCT independently predicted intensive care unit (ICU) death. PCT provided no predictive value beyond existing inflammatory markers, consistent with its suppression during viral infections. **Conclusions**: Day 5 ferritin reflects, rather than independently predicts, organ dysfunction severity and may complement, rather than replace, established multi-parameter scoring. Relative to the independent determinants of severity and mortality (PaO_2_/FiO_2_ ratio, LDH, CRP, and age), day 5 ferritin is a specific, rule-in adjunctive marker of concurrent organ dysfunction rather than a standalone prognostic tool. Whether these associations extend to non-COVID sepsis populations requires prospective study.

## 1. Introduction

In December 2019, severe acute respiratory syndrome coronavirus 2 (SARS-CoV-2) emerged in Wuhan, China, causing a novel disease later termed coronavirus disease 2019 (COVID-19). The infection spread rapidly worldwide and was declared a pandemic by the World Health Organization (WHO) in March 2020, coinciding with the admission of critically ill patients to intensive care units (ICUs) across Europe [1]. Patients with COVID-19 requiring ICU care typically present with progressive respiratory failure secondary to severe pulmonary involvement. In addition to respiratory compromise, SARS-CoV-2 affects extrapulmonary systems, including the liver, kidneys, and haematological components, thereby increasing the risk of multi-organ failure [2]. The virus can infect both ciliated and secretory epithelial cells in the respiratory tract, contributing to its high transmissibility and pathogenicity compared with other coronaviruses [3].

In critically ill patients, clinical scoring systems support the assessment of disease severity and guide therapeutic decision-making [4]. The Sepsis-related Organ Failure Assessment (SOFA)-2 score evaluates the extent of organ dysfunction across multiple systems and may provide valuable prognostic information regarding disease progression and mortality risk, making it a practical tool for risk stratification at admission and during daily follow-up of critically ill patients with COVID-19 and COVID-19-associated sepsis. Previous work by Raschke et al. reported that the SOFA-2 score did not predict mortality in patients with COVID-19 pneumonia requiring mechanical ventilation [5]; however, their analysis was limited to a specific patient subset. In contrast, our study categorised patients according to disease severity and included a broader ICU population, enabling the association between the SOFA-2 score and mortality across severity groups to be evaluated.

Cytokine storm, an uncontrolled immune response, plays a central role in the immunopathogenesis of COVID-19, characterised by excessive release of pro-inflammatory cytokines, including tumour necrosis factor-α (TNF-α), interleukin (IL)-6, IL-12, and IL-8, which may lead to acute respiratory distress syndrome (ARDS) and multi-organ failure [6]. Emerging evidence indicates that biomarkers such as serum ferritin (ng/mL), D-dimer, lactate dehydrogenase (LDH; U/L), and IL-6 increase with disease progression and are associated with higher mortality risk [7]. In particular, hyperferritinaemia, reflecting severe inflammation, has been linked to ICU admission and increased mortality and may serve as a useful marker for identifying high-risk patients and guiding anti-inflammatory therapeutic strategies [8,9,10]. Hyperferritinemia is a hallmark of haemophagocytic lymphohistiocytosis (HLH), a recognised complication of viral infections, and is closely associated with poor outcomes in patients with COVID-19 [11]. In adult-onset Still’s disease (AOSD), elevated ferritin levels are linked to macrophage activation syndrome (MAS), the most frequent life-threatening complication, and are associated with higher mortality [12]. Similarly, in sepsis, elevated ferritin levels correlate with greater disease severity and poorer prognosis [13], supporting the concept of a hyperferritinaemic syndrome shared across inflammatory conditions [14]. Ferritin production by the liver and macrophages is upregulated during inflammatory states, driven by cytokines such as IL-1β, IL-6, and interferon (IFN)-γ [15]. Given its proposed role in immune activation, targeting ferritin or its regulatory pathways may represent a potential therapeutic strategy in hyperinflammatory conditions, including severe COVID-19 [16,17].

Several biomarkers have been proposed to predict disease severity in COVID-19, among which PCT (μg/L) is frequently investigated [18]. PCT, the prohormone of calcitonin, is normally produced by thyroid C cells under physiological conditions. During systemic inflammation, its production is upregulated in multiple tissues. Although elevated PCT levels are typically associated with bacterial infections, their synthesis can also be triggered by inflammatory mediators such as IL-6 and TNF-α, which are increased in severe COVID-19 [19]. In patients with COVID-19 and pulmonary involvement such as ARDS, PCT may reflect the degree of hyperinflammation [20] and can assist in identifying concurrent bacterial infections, supporting antibiotic stewardship decisions [21,22,23]. However, studies on isolated viral COVID-19, similar to other viral infections, have shown that PCT often remains within normal limits (≤0.5 μg/L), possibly due to viral suppression of TNF-α through macrophage-mediated IFN-γ production [23]. As an early biomarker of systemic inflammation [24], PCT has been linked to prognosis in infection-related diseases and has been identified as an independent risk factor in sepsis. Several studies have also reported associations between elevated PCT and COVID-19 severity [25,26,27].

**Figure 1:**
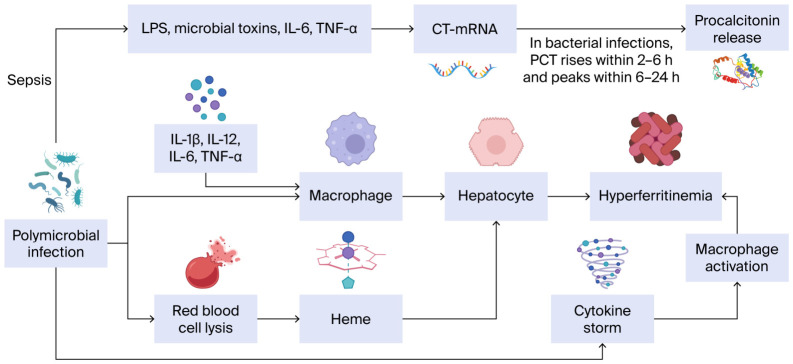
Pathophysiological mechanism of hyperferritinaemia and elevated PCT in sepsis. IL, interleukin; TNF-α, tumour necrosis factor-α; LPS, lipopolysaccharide; PCT, procalcitonin.

Macrophages play a central role in sepsis through pathogen recognition, signalling pathway activation, cytokine production, and immunomodulation. Using surface toll-like receptors (TLRs), macrophages detect pathogen-associated molecular patterns and activate the TLR4/MyD88/NF-κB signalling cascade, leading to the robust release of pro- and anti-inflammatory cytokines. Figure 1 depicts the pathophysiological mechanisms underlying hyperferritinaemia and elevated PCT in sepsis [28].

In summary, this study aimed to evaluate the relationship among serum ferritin (ng/mL), serum PCT (μg/L), and the SOFA-2 score in patients with COVID-19-associated sepsis and, through exploratory analysis, to examine their relationship with ICU mortality.

## 2. Materials and Methods

### 2.1. Study Design and Setting

This retrospective study included patients treated in the COVID-19 ICU at Afyonkarahisar University of Health Sciences Hospital who were diagnosed with COVID-19-associated sepsis and received favipiravir therapy between 1 March 2020 and 30 June 2021. COVID-19 diagnosis was confirmed via SARS-CoV-2 PCR-RNA testing using nasopharyngeal swabs or bronchoalveolar lavage (BAL) samples. The SOFA-2 score was calculated for all patients retrospectively in 2026 by re-applying the SOFA-2 criteria published by Ranzani et al. [29] to clinical and laboratory variables prospectively recorded during routine ICU care in 2020–2021. The SOFA-2 score is the 2025 revision of the original 1996 SOFA score [29]; although both scores grade dysfunction across the respiratory, cardiovascular, hepatic, coagulation, renal, and neurological systems, SOFA-2 updates and redistributes the organ-system thresholds to account for monitoring and organ-support interventions that were unavailable when the original score was devised, with the stated aim of improving content validity and interpretability in contemporary critical care. All variables required for SOFA-2 calculation (respiratory, cardiovascular, hepatic, coagulation, renal, and neurological domains) were available from the electronic medical record; those with a score of <2 were excluded. Ferritin (ng/mL) and PCT (μg/L) levels were evaluated in patients with an SOFA-2 score of ≥2.

**Figure 2:**
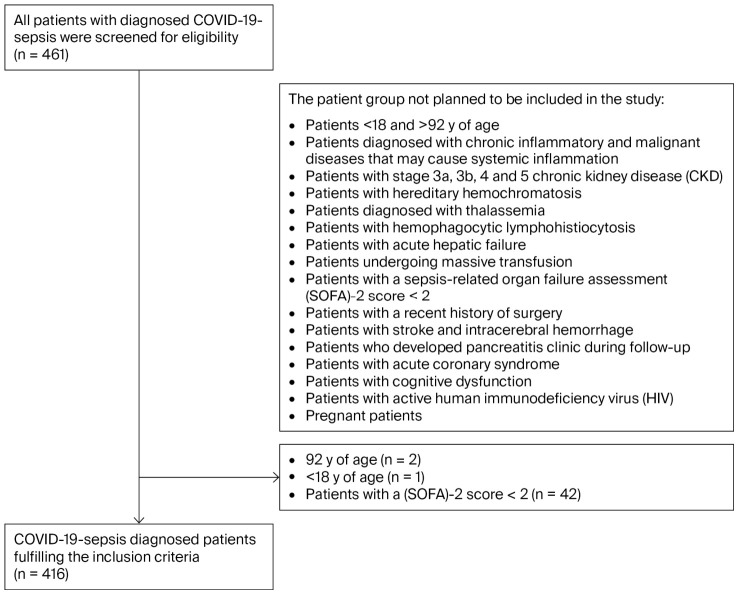
Exclusion criteria flowchart.

Data from 461 patients meeting the inclusion criteria were initially screened. A total of 45 patients were subsequently excluded: 2 patients aged 92 years, 1 patient aged under 18 years, and 42 patients with an SOFA-2 score below 2. Ultimately, 416 patients were included in the analysis. The flowchart for exclusion criteria is presented in Figure 2.

The COVID-19 disease severity index was developed based on the WHO’s classification of COVID-19-related clinical syndromes. Sepsis is defined as life-threatening organ dysfunction caused by a dysregulated host response to a suspected or confirmed infection, with key indicators including altered mental status, tachypnea, hypoxemia, oliguria, tachycardia, hypotension, peripheral circulatory abnormalities, coagulopathy, thrombocytopenia, hyperlactatemia, and hyperbilirubinemia [30].

Patient data were extracted from electronic medical records. Laboratory data collected at admission included fasting plasma glucose (mmol/L), serum creatinine (μmol/L), estimated glomerular filtration rate (eGFR; mL/min/1.73 m^2^), white blood cell count (WBC; /μL) with differential, haemoglobin (g/dL), platelets (/μL), C-reactive protein (CRP; mg/dL), PCT (μg/L), ferritin (ng/mL), erythrocyte sedimentation rate (ESR; mm/h), D-dimer (μg/mL), ALT and AST (U/L), total and indirect bilirubin (μmol/L), electrolytes (mmol/L), albumin (g/dL), serum lactate (mmol/L), and PaO_2_/FiO_2_ ratio (mmHg). The same parameters were collected on day 5. Antibiotic treatment data were not included because bacterial infections were not present in all patients and treatment protocols varied. Additional parameters that were recorded (electrolytes, AST, ALT, bilirubin, albumin, lactate, and ESR) were used for clinical characterisation and, where applicable such as bilirubin, for SOFA-2 computation; because they were not among the pre-specified biomarkers of interest, they are not reported individually.

PCT levels were measured using the E801 Elecsys BRAHMS PCT assay on the COBAS 8000 system (Roche Diagnostics, Rotkreuz, Switzerland). Patients were grouped based on PCT levels as <2, 2–10, and ≥10.01 μg/L, using established cut-off thresholds [31]. Ferritin levels (ng/mL) were categorised as 0–499, 500–1000, and ≥1001 ng/mL. Due to the 80 patients who died or were discharged before day 5 and lacked day 5 measurements, analysis was restricted to the 336 patients with available data. Plasma ferritin (ng/mL) was measured using the ECLIA method on the Cobas e601 analyzer (Roche Diagnostics, Rotkreuz, Switzerland). Patients with hereditary haemochromatosis or CKD (eGFR < 60 mL/min/1.73 m^2^) were excluded. An SOFA-2 score cut-off of ≥5 was used to classify high severity, consistent with the severity stratification framework of the SOFA-2 validation study by Ranzani et al. [29] and applied in COVID-19 ICU cohort studies [32].

### 2.2. Statistical Analysis

Continuous variables are presented as the mean ± SD for normally distributed data (Shapiro–Wilk test) and as the median (IQR) for non-normal data. Categorical variables are expressed as counts and percentages. Normally distributed variables were compared using Student’s *t*-test, non-normally distributed variables with the Mann–Whitney U test, and groups of three or more with the Kruskal–Wallis test, with the results reported as the median (IQR). Categorical variables were compared using the chi-squared or Fisher’s exact test. Multivariate logistic regression was performed using a stepwise model (entry probability 0.05). To address the recognised instability of stepwise selection, the primary multivariable models were re-estimated using the forced entry of all pre-specified covariates, and multicollinearity was assessed using variance inflation factors (VIFs). The robustness of the day 5 ferritin association was further examined across alternative SOFA-2 severity thresholds (≥4, ≥5, and ≥6) and against the continuous score using Spearman’s correlation. A separate multivariable logistic regression model was constructed with ICU mortality as the dependent variable, using admission biomarkers, available for all patients, to mitigate survivorship bias, with adjustments made regarding the PaO_2_/FiO_2_ ratio, LDH, CRP, haemoglobin, age, sex, comorbidities, and treatment. The proportion of missing data was quantified for every analysed variable; because day 5 values were unavailable for patients who died or were discharged before day 5, analyses involving day 5 markers were performed on a complete-case basis. ROC curve analysis was used to evaluate ferritin’s diagnostic accuracy, with the AUC calculated and the Youden index applied to determine optimal cut-off values. Model calibration was assessed using the Hosmer–Lemeshow test. Given the exploratory nature of multiple comparisons across biomarker subgroups, results are interpreted in the context of the overall model rather than by applying post hoc correction; findings with a *p* < 0.05 are considered statistically significant and are presented as ORs with 95% CIs. Statistical analyses were performed using SPSS version 25.0 (SPSS Inc., Chicago, IL, USA).

### 2.3. Ethics Approval

This study was conducted in accordance with the 1964 Declaration of Helsinki and its later amendments. Patient data were originally collected during routine clinical care between March 2020 and June 2021. The present retrospective analysis, which included re-calculation of the SOFA-2 score using its 2025 definition [29], was reviewed and approved by the Afyonkarahisar Health Sciences University Clinical Research Ethics Committee (decision date: 6 March 2026; meeting number: 2026/3) under the framework for the retrospective use of de-identified clinical data. Informed consent was waived by the ethics committee for this retrospective study. Clinical trial registration was not applicable.

## 3. Results

A total of 416 patients were included in the study. Of these, 284 (68.3%) were male and 132 (31.7%) were female, with a median age of 69 years (IQR: 62–76). Overall, 287 patients (69.0%) had an SOFA-2 score of 0–4 and 129 patients (31.0%) had a score ≥ 5. Patient characteristics, clinical outcomes, comorbidities, and treatments stratified by SOFA-2 score group are presented in Table 1. Mean laboratory parameters for the entire cohort are summarised in Table 2. The overall mean PCT at admission was 2.4 ± 11.34 μg/L, increasing to 3.6 ± 13.4 μg/L on day 5. Antiviral therapy consisted of favipiravir; no patient received remdesivir, and targeted immunomodulatory therapy was administered to only 12 patients (tocilizumab, *n* = 3; anakinra, *n* = 9; 2.9%), whereas systematic data on corticosteroid administration were not available. The study period (March 2020–June 2021) preceded the widespread availability of COVID-19 vaccination in the source population, and individual vaccination status was not recorded in the medical records; the cohort therefore predates routine vaccination.

**Figure 3:**
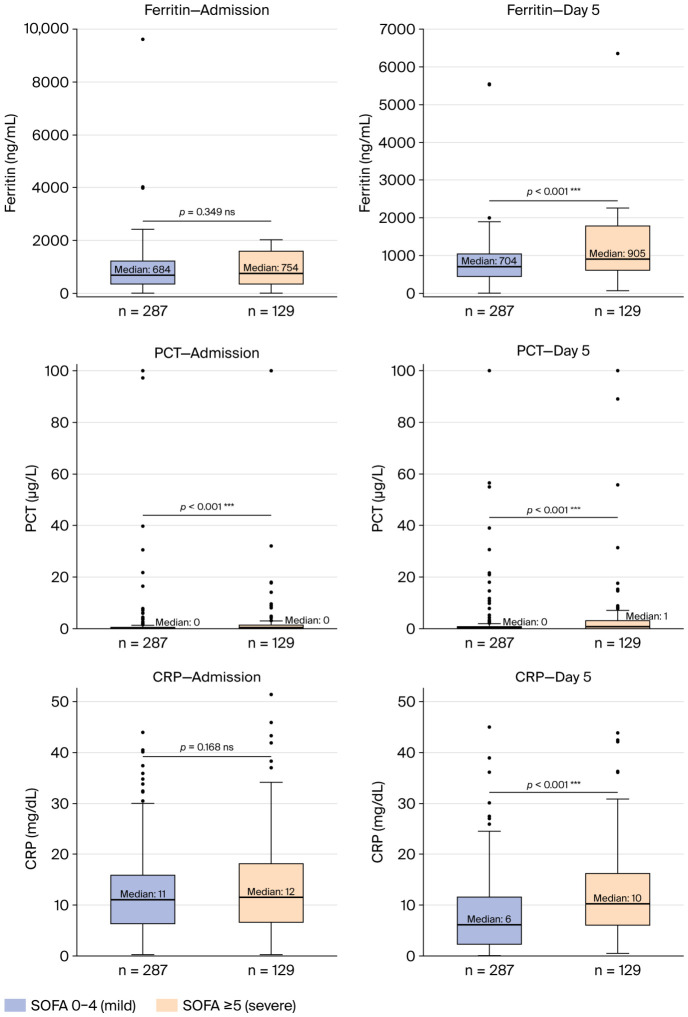
Biomarker distribution by SOFA-2 score severity group. Box plots show median and IQR for ferritin (ng/mL), PCT (μg/L), and CRP (mg/dL) at admission and on day 5. *** *p* < 0.001, ns = not significant (Mann–Whitney U test).

ICU mortality was significantly higher in the severe group: 44.3% (SOFA-2 0–4) versus 86.8% (SOFA-2 ≥ 5) (*p* < 0.001). Mechanical ventilation was required by 31.0% and 65.1% of patients in the mild and severe groups, respectively (*p* < 0.001). The severe group also had substantially higher rates of renal replacement therapy (23.3% vs. 7.3%; *p* < 0.001) and greater burden of heart failure, CKD, and ASCVD (Table 1). Day 5 ferritin was 854 ng/mL in the mild group versus 1110 ng/mL in the severe group (*p* = 0.001), and day 5 CRP was 8.11 versus 12.62 mg/dL (*p* < 0.001). The distribution of key biomarkers across severity groups at admission and day 5 is illustrated in Figure 3 (box plots).

The laboratory parameters by SOFA-2 score group are shown in Table 3. Patients with SOFA-2 ≥ 5 had significantly higher day 5 PCT (6.44 ± 19.06 vs. 2.23 ± 9.31 μg/L; *p* < 0.001), day 5 ferritin (1110 ± 816 vs. 854 ± 682 ng/mL; *p* = 0.001), and day 5 CRP (12.62 ± 9.25 vs. 8.11 ± 7.67 mg/dL; *p* < 0.001), while their PaO_2_/FiO_2_ ratio (111 ± 24 vs. 126 ± 35 mmHg; *p* < 0.001) and haemoglobin levels (12.03 ± 2.28 vs. 12.58 ± 2.04 g/dL; *p* = 0.009) were significantly lower.

Among comorbidities, heart failure (38.0% vs. 20.6%; *p* < 0.001), CKD (14.7% vs. 6.3%; *p* = 0.005), and ASCVD (34.9% vs. 19.5%; *p* = 0.001) were significantly more prevalent in the SOFA-2 ≥ 5 group (Table 4).

Multivariate logistic regression analyses evaluating the independent associations of day 5 PCT and day 5 ferritin with SOFA-2 score are presented in Table 5. In Model A, day 5 PCT added in Step 2 was not a statistically significant predictor (OR = 1.001; 95% CI 0.981–1.022; *p* = 0.887), despite the significance of the PaO_2_/FiO_2_ ratio, LDH, day 5 CRP, ASCVD, and hydroxychloroquine in Step 1. In Model B, day 5 ferritin added in Step 2 remained a statistically significant independent predictor (OR = 1.000 per unit; 95% CI 1.000–1.001; *p* = 0.014), above and beyond the PaO_2_/FiO_2_ ratio (*p* = 0.004), LDH (*p* = 0.005), day 5 CRP (*p* = 0.002), and hydroxychloroquine use (*p* = 0.014).

**Figure 4:**
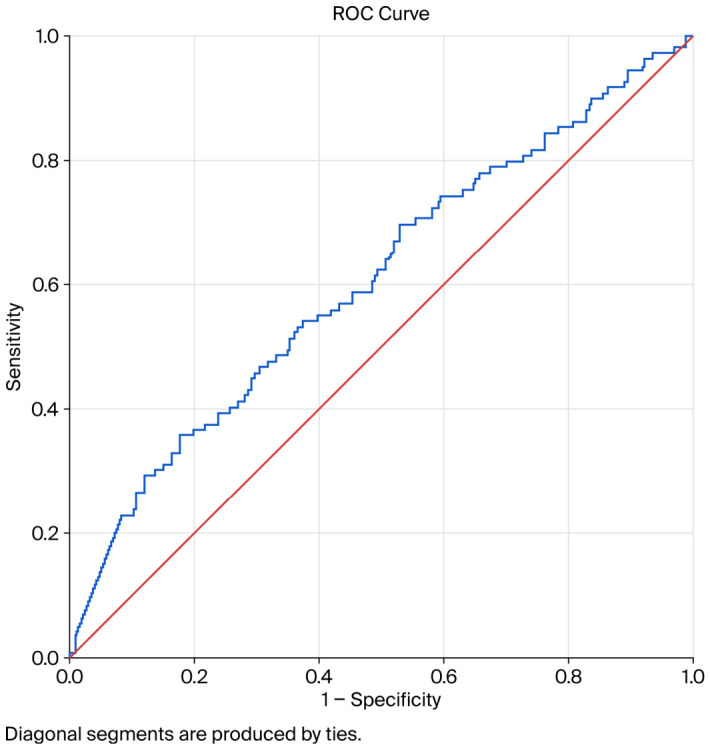
ROC curve of day 5 ferritin. The blue line represents the ROC curve for day 5 ferritin, and the red diagonal indicates the reference line (line of no discrimination, AUC = 0.5).

On day 5, ROC curve analysis of ferritin showed an AUC of 0.608 (SE = 0.034), with an optimal cut-off of >1191 ng/mL (sensitivity 35.78%, 95% CI 26.8–45.5; specificity 82.38%, 95% CI 76.8–87.1) (Table 6; Figure 4).

The laboratory parameters across day 5 PCT subgroups are presented in Table 7. Patients with PCT ≥ 10.01 μg/L had significantly lower day 5 lymphocyte counts [488 (228–836) vs. 752 (374–1108) /μL; *p* = 0.027], and significantly higher day 5 ferritin [1102 (704–1568) vs. 724 (320–1256) ng/mL; *p* = 0.001] and day 5 CRP [14.6 (8.4–24.2) vs. 6.4 (2.8–11.4) mg/dL; *p* < 0.001]. No significant association was found between the day 5 PCT group and chronic disease or treatment parameters (Table 8).

Laboratory parameters across day 5 ferritin subgroups are presented in Table 9. Patients with ferritin ≥ 1001 ng/mL had significantly higher LDH [578 (388–836) vs. 398 (280–560) U/L; *p* < 0.001], day 5 WBC [14,280 (8660–20,480) vs. 9860 (6880–13,200) /μL; *p* < 0.001], and day 5 CRP [10.0 (4.4–18.6) vs. 5.6 (2.0–10.6) mg/dL; *p* < 0.001]. No significant association with chronic disease or treatment parameters was found (Table 10).

### Mortality Model, Survivorship, and Robustness Analyses

In a dedicated multivariable logistic regression model with ICU mortality as the dependent variable, using admission biomarkers available for all 416 patients, neither ferritin (OR per 100 ng/mL 1.003, 95% CI 0.971–1.035; *p* = 0.87) nor PCT (OR 0.998, 95% CI 0.978–1.019; *p* = 0.88) was an independent predictor of death. The independent predictors of ICU mortality were a lower PaO_2_/FiO_2_ ratio (OR 0.980 per mmHg, 95% CI 0.972–0.988; *p* < 0.001), higher LDH (OR 1.199 per 100 U/L, 95% CI 1.087–1.321; *p* < 0.001), higher CRP (OR 1.032 per mg/dL, 95% CI 1.005–1.059; *p* = 0.018), and older age (OR 1.033 per year, 95% CI 1.012–1.053; *p* = 0.001).

To assess survivorship bias arising from the exclusion of patients without day 5 measurements, we repeated the biomarker–severity analysis using admission values, which were available for the entire cohort. Admission ferritin did not differ between severity groups (median 684 vs. 754 ng/mL; *p* = 0.35; AUC 0.53), whereas day 5 ferritin did (median 704 vs. 905 ng/mL; *p* < 0.001; AUC 0.61). Admission PCT discriminated severity groups (AUC 0.61; *p* < 0.001), and day 5 PCT did so more strongly (AUC 0.71; *p* < 0.001). Patients lacking day 5 data had a higher in-ICU mortality (60.8%) than the cohort overall, consistent with selective loss of the most severely ill patients.

To further characterise potential survivorship bias, we compared the 336 patients with available day 5 data with the 80 patients without such data (who died or were discharged before day 5) in terms of admission characteristics. The two groups were similar in age (median 68 vs. 70 years; *p* = 0.88), sex, and the prevalence of hypertension, diabetes, heart failure, coronary artery disease, and chronic kidney disease (all *p* > 0.19); critically, they did not differ in admission ferritin (median 717 vs. 632 ng/mL; *p* = 0.34), admission PCT (*p* = 0.88), or admission CRP (*p* = 0.80). Patients without day 5 data had a lower admission SOFA-2 score (median 3 vs. 4; *p* < 0.001), lower LDH (391 vs. 470 U/L; *p* = 0.001), and a higher PaO_2_/FiO_2_ ratio (132 vs. 110 mmHg; *p* < 0.001), reflecting that this group comprised both early deaths and patients discharged early after clinical improvement; malignancy was somewhat more frequent among them (20.3% vs. 10.4%; *p* = 0.02). The comparable admission ferritin between the groups indicates that the day 5 ferritin–severity association is unlikely to be an artefact of the selective loss of patients with high baseline ferritin.

The association between day 5 ferritin and SOFA-2 severity was directionally stable across alternative dichotomisation thresholds (AUC 0.57, 0.61, and 0.61 for SOFA-2 ≥ 4, ≥5, and ≥6, respectively) and against the continuous score (Spearman ρ = 0.16; *p* = 0.003), supporting use of the ≥5 cut-off while indicating a weak overall correlation. Multicollinearity among the day 5 covariates was negligible (all variance inflation factors between 1.0 and 1.2). When the model was re-estimated considering forced entry, day 5 ferritin was not an independent correlate of SOFA-2 severity (*p* = 0.23), although it was significant in the univariable analysis (*p* = 0.004); stepwise and forced-entry approaches yielded concordant conclusions regarding the absence of an independent ferritin effect.

## 4. Discussion

This retrospective study evaluated the relationship of serum ferritin (ng/mL) and PCT (μg/L) with SOFA-2 score-based organ dysfunction severity in patients with COVID-19-associated sepsis. Our central findings are as follows: (i) Day 5 serum ferritin was associated with concurrent SOFA-2 severity in the univariable analysis and across alternative severity thresholds, but did not remain an independent correlate once day 5 CRP and LDH were entered together in a forced-entry model. (ii) Day 5 PCT discriminated severity groups in the univariable analysis yet provided no independent information after adjustment, a finding that is biologically coherent with PCT’s known suppression during primary viral infections. (iii) In a dedicated logistic regression model for ICU mortality, neither ferritin nor PCT was an independent predictor of death, whereas the PaO_2_/FiO_2_ ratio, LDH, CRP, and age were. Because both biomarkers and the SOFA-2 score were assessed contemporaneously on day 5, these results describe a cross-sectional association with concurrent organ dysfunction rather than the prediction of subsequent outcomes. This is one of the first studies to simultaneously examine the relationship between both biomarkers and the SOFA-2 score in patients specifically diagnosed with COVID-19-associated sepsis. Previous studies have explored a range of clinical and laboratory variables to improve risk assessment in critically ill patients with COVID-19, including D-dimer, prothrombin time, haematological indices, and ferritin, but none has simultaneously evaluated both PCT and ferritin against SOFA-2 score in a sepsis-defined ICU cohort [33,34,35,36].

### 4.1. Ferritin and Organ Dysfunction Severity

The predictive value of ferritin has been established for sepsis severity and outcomes. Nonlinear associations between serum ferritin and mortality have been described, with each 1000 ng/mL increment corresponding to higher mortality rates at 28 days, 90 days, and 1 year [37]. Elevated ferritin is also associated with an increased incidence of sepsis [38], and levels > 1000 ng/mL have been linked to hyperinflammatory states, including sepsis, HLH, and MAS [39]. Under physiological conditions, serum ferritin ranges from 30 to 300 ng/mL in men and 10 to 200 ng/mL in women, with a plasma half-life of approximately 30 h; its circulating levels rise markedly during viral infections and may reflect viral replication kinetics [40,41,42].

In our cohort, day 5 ferritin was significantly lower in patients with SOFA-2 0–4 (854 ± 682 ng/mL) than in those with SOFA-2 ≥ 5 (1110 ± 816 ng/mL; *p* = 0.001). In the univariable analysis, day 5 ferritin discriminated severity groups (AUC 0.608; univariable *p* = 0.004), and the association was directionally consistent across alternative SOFA-2 thresholds (≥4, ≥5, ≥6) and against the continuous score (Spearman ρ = 0.16; *p* = 0.003). However, when day 5 CRP and LDH were entered together with ferritin in a forced-entry model, ferritin no longer remained an independent correlate of SOFA-2 score (adjusted *p* = 0.23), indicating that it adds little discriminatory information beyond these established inflammatory and tissue injury markers; the result was unchanged in a stepwise model, and multicollinearity was not responsible (all variance inflation factors < 1.2). Although both are acute-phase reactants, their induction pathways differ: CRP is a rapid, IL-6-driven hepatic response, whereas circulating ferritin originates predominantly from activated macrophages and reflects iron-mediated immunopathology, including MAS, oxidative tissue damage, and ferroptosis, through mechanisms that are at least partially CRP-independent [43,44].

The timing of day 5 measurement is clinically relevant. Day 5 represents a transitional phase between initial viral replication and the immune-mediated inflammatory amplification driving severe COVID-19. Ferritin levels at this point capture both the initial inflammatory response and the evolving cytokine storm. Consistent with the literature, the optimal cut-off of >1191 ng/mL showed a high-specificity, low-sensitivity profile (specificity 82.38%; sensitivity 35.78%), positioning ferritin as a rule-in rather than rule-out marker [45,46,47]. The development of ARDS, the primary cause of death when COVID-19 progresses to respiratory failure, is driven by cytokine storm and exaggerated host immune responses, in which elevated ferritin plays a central role [48].

These findings are consistent with the broader concept of a hyperferritinaemic syndrome shared across AOSD, MAS, sepsis, and catastrophic antiphospholipid syndrome [14]. SARS-CoV-2 provokes macrophage activation through the release of IL-6, IL-1β, and TNF-α, which directly stimulate ferritin synthesis; emerging evidence further implicates ferroptosis, an iron-dependent cell death pathway, in multi-organ injury, with ferroptosis markers correlating with lung injury severity [44]. Ferritin’s association with the SOFA-2 score is consistent with systemic macrophage activation and iron-mediated injury contributing to organ-level dysfunction beyond the lungs, although this association did not persist as an independent effect after adjustment.

### 4.2. PCT and SOFA-2 Score: Biological Context of a Negative Finding

After engaging Toll-like receptors, SARS-CoV-2 triggers an inflammatory cascade through pro-inflammatory cytokines such as IL-1 and IL-6, which can upregulate PCT production [49]. In severe COVID-19, cytokine storm and immunological hyperactivation may elevate PCT even in the absence of bacterial co-infection [50]. Nevertheless, PCT synthesis is typically induced by bacterial lipopolysaccharide and cytokines, chiefly IL-1β and TNF-α, but is physiologically suppressed during viral infections by IFN-γ. Consistent with findings from prior coronavirus epidemics (SARS, MERS) and influenza H1N1, PCT remains ≤0.5 μg/L in most patients with uncomplicated viral COVID-19, rising primarily in severe cases or fatal outcomes [51,52,53]. Meta-analytic evidence has reported a positive association between elevated PCT and increased risk of severe COVID-19 [54], yet this elevation reflects secondary bacterial superinfection or extreme hyperinflammation rather than the primary viral inflammatory cascade.

In the univariable analysis, day 5 PCT discriminated severity groups reasonably well (AUC 0.71); however, in multivariate logistic regression, neither baseline nor day 5 PCT remained a significant correlate of SOFA-2 score after adjustment. PCT’s non-significance arose in a model that already included day 5 CRP (*p* = 0.004) and LDH, biomarkers capturing the bulk of the virally driven inflammatory signal. This statistical attenuation within a comprehensive model does not imply that PCT lacks biological relevance; rather, it indicates that PCT does not provide independent discriminatory information for organ dysfunction severity beyond what is already conveyed by these markers in COVID-19-associated sepsis. This interpretation is consistent with reports showing that PCT does not reliably identify bacterial co-infection or independently stratify viral disease severity in this population [55,56], although some observational studies have reported an association between elevated PCT and in-hospital mortality in specific COVID-19 cohorts [57,58]. The age-dependent variation in PCT’s predictive performance is also relevant for hospitalised elderly cohorts [56]. Clinically, PCT remains most valuable in its primary validated role: guiding antibiotic stewardship and identifying secondary bacterial complications in patients whose deterioration exceeds what the viral illness alone would explain. Moreover, because systematic data on bacterial co-infection and antibiotic exposure were not available, the biological meaning of an individual elevated PCT value cannot be fully resolved; therefore, our finding of no independent association should not be considered evidence that PCT is uninformative in this setting.

### 4.3. Clinical Implications and Applicability Beyond COVID-19

Although day 5 ferritin was not an independent correlate of SOFA-2 score after adjustment, its consistent univariable association with organ dysfunction severity carries more modest, hypothesis-generating clinical implications. First, it is compatible with monitoring serial ferritin, particularly around day 5, within broader COVID-19 assessment, but only as one component of a multi-marker evaluation rather than as a standalone tool. Second, ferritin may behave as a dynamic marker of the inflammatory trajectory: a lack of decline by day 5 could reflect ongoing macrophage activation, a hypothesis that would require prospective testing before it could be applied to guide immunomodulatory decisions such as on corticosteroid or IL-6 pathway inhibition. Third, because ferritin is inexpensive and widely available, it may have value as an adjunctive severity indicator in resource-limited settings, provided that its limited standalone discrimination (AUC 0.61) and lack of independent predictive value are explicitly recognised.

The magnitude of the day 5 ferritin effect also warrants cautious clinical interpretation. The adjusted odds ratio of approximately 1.000 per ng/mL should be read in the context of the measurement unit: expressed per 1000 ng/mL it corresponds to an odds increase of roughly 1.5-fold, so the effect is not biologically negligible, yet on a per-unit basis the incremental information is small. Taken together with an AUC of 0.608 and a sensitivity of 35.78%, these metrics indicate that day 5 ferritin adds little discriminative value beyond routinely available, inexpensive markers such as CRP and LDH and beyond established multi-parameter severity scores. Accordingly, a meaningful net clinical benefit from using day 5 ferritin as a standalone screening or risk-stratification tool cannot be inferred from the present data; its role is best regarded as a specific, rule-in adjunct that complements, rather than improves upon, existing risk-assessment approaches.

The applicability of these findings extends beyond the COVID-19 population studied. Elevated ferritin in COVID-19 shares a common pathophysiological substrate with hyperferritinaemic states in non-COVID bacterial sepsis, MAS, and septic shock, all characterised by dysregulated macrophage activation, excessive cytokine release, and iron-mediated immunopathology. Understanding the pathogenesis of sepsis and identifying therapeutic targets, including immunomodulatory approaches, remains an active area of research that may further clarify the role of ferritin and related pathways in clinical outcomes [59,60,61,62]. In non-COVID sepsis populations, ferritin has consistently emerged as an independent predictor of 28-day mortality with nonlinear dose–response relationships [37,46]. The parallel finding that PCT provides limited independent predictive value for organ dysfunction severity within a comprehensive inflammatory marker panel may also have relevance in mixed ICU populations. Clinicians managing sepsis of any aetiology may therefore consider serial ferritin measurement as a complement to existing organ dysfunction scoring systems, interpreting PCT primarily in the context of suspected bacterial co-infection rather than as a standalone severity marker.

### 4.4. Limitations

Several limitations should be acknowledged. First, the retrospective design limits causal inference. Second, as a retrospective study, a formal a priori power calculation was not performed; the sample size of 416 reflects real-world clinical availability. The adequacy of statistical power for the primary multivariate analyses is supported by the observation of significant effects, but subgroup analyses should be interpreted with appropriate caution. Third, day 5 ferritin and PCT values were unavailable for 80 patients who died or were discharged before day 5 (19.2% of the cohort), introducing potential survivorship bias; contrary to a purely conservative effect, sensitivity analysis using admission values, available for all patients, revealed no association between admission ferritin and SOFA-2 severity (*p* = 0.35), and the ferritin–severity association emerged only at day 5. The day 5 finding may therefore partly reflect selective survival of less severely affected patients rather than a robust biomarker effect. Formal multiple imputation was not performed; analyses are therefore restricted to the 336 patients with available day 5 data. Fourth, antibiotic treatment data were not systematically included because bacterial infections were not present in all patients and no standardised regimen was administered. Fifth, patients with hereditary haemochromatosis or CKD (eGFR < 60 mL/min/1.73 m^2^) were excluded, limiting generalisability to these subgroups. Sixth, given the exploratory nature of multiple comparisons across biomarker subgroups, our results should be interpreted in the context of the overall model, and prospective validation in independent cohorts is warranted. Seventh, the SOFA-2 score was applied retrospectively in 2026 to data collected before the score’s 2025 publication [29]. Although all variables required for SOFA-2 score calculation were available in the original electronic medical records, prospective application of the score may yield slightly different operating characteristics, and our findings should be re-examined in cohorts in which SOFA-2 is collected prospectively. Eighth, the diagnostic performance of day 5 ferritin at the optimal cut-off (>1191 ng/mL) was modest (AUC 0.608; sensitivity 35.78%), reflecting a high-specificity, low-sensitivity profile. This indicates that ferritin elevation above this threshold is informative as a rule-in marker for severe organ dysfunction but cannot, in isolation, rule out severity in patients with lower ferritin levels; clinical use should therefore be as an adjunct to, not a replacement for, established multi-parameter scoring systems. Ninth, all included patients received favipiravir as part of contemporary local treatment protocols; generalisability to cohorts treated with other antiviral or immunomodulatory regimens (e.g., remdesivir, tocilizumab, baricitinib, or dexamethasone-based protocols) requires further validation, particularly given the substantial evolution of standard care during and after the study period. Finally, while the study period (2020–2021) captures an important phase of the pandemic, treatment protocols have evolved substantially, and findings may not be fully generalisable to later variant epochs. In addition, because both biomarkers and the SOFA-2 score were measured contemporaneously on day 5, the design is cross-sectional and cannot lead to temporal or prognostic precedence; the principal day 5 ferritin association, although significant in the univariable analysis, did not persist under forced-entry adjustment, underscoring that it should be regarded as a correlate of concurrent severity rather than an independent marker. A time-to-event framework, such as Cox proportional-hazards or competing-risk modelling, was considered as a means of accounting for early mortality. However, because day 5 ferritin and the day 5 SOFA-2 score are ascertained at the same cross-sectional time point, the primary association has no time-to-event structure, and such models are not directly applicable to it. The informative loss of patients before day 5 was instead addressed through a mortality model restricted to admission biomarkers available for all 416 patients and through a direct comparison of the included and excluded groups, neither of which indicated that early loss had materially inflated the day 5 ferritin findings. Formal competing-risk or joint longitudinal-survival modelling would require prospectively and serially sampled cohorts, and it represents a valuable direction for future validation. Finally, one implausible day 5 haemoglobin value was identified as a data entry error and corrected prior to the present analyses. Additionally, body mass index was not available in the retrospective records and could not be included among the baseline covariates. Corticosteroid and other immunomodulator administration was likewise not systematically recorded, precluding adjustment for these potential confounders; reassuringly, targeted immunomodulators (tocilizumab and anakinra) were used in only 2.9% of the cohort, limiting their likely confounding influence on ferritin and CRP. In addition, detailed COVID-19 vaccination status was unavailable, although the study period largely preceded population-level vaccination.

## 5. Conclusions

Day 5 serum ferritin was associated with concurrent SOFA-2 score-based organ-dysfunction severity in patients with COVID-19-associated sepsis in the univariable analysis, but did not remain an independent correlate after adjustment for CRP and LDH and did not independently predict ICU mortality. Therefore, it appears to reflect, rather than independently predict, disease stratification. A cut-off of >1191 ng/mL provides high specificity (82.38%) but modest sensitivity (35.78%; AUC 0.608), positioning it as a rule-in rather than rule-out descriptor of severity. Values above this threshold are relatively specific for severe organ dysfunction, whereas lower values do not exclude it. Given its limited standalone discrimination, ferritin is best interpreted alongside, rather than in place of, established multi-parameter scores. In contrast, PCT did not provide independent predictive value for organ dysfunction severity in this context, consistent with its known suppression during primary viral infection; its primary utility remains in guiding antibiotic stewardship and detecting secondary bacterial complications. These findings support the consideration of serial ferritin measurement as a complement to established organ dysfunction scoring in COVID-19 ICU monitoring protocols, pending prospective validation, and suggest broader relevance in sepsis management where macrophage activation and iron dysregulation contribute to organ failure.

## Figures and Tables

**Table 1:** Patient characteristics by SOFA-2 score group.

Characteristic	Total (*n* = 416)	SOFA-2 0–4 (*n* = 287)	SOFA-2 ≥ 5 (*n* = 129)	*p*-Value
Demographics				
Age, years [median (IQR)]	69 (62–76)	68 (60.5–76)	69 (65–75)	0.204
Male sex, *n* (%)	284 (68.3%)	193 (67.2%)	91 (70.5%)	0.580
Clinical outcomes				
ICU mortality, *n* (%)	239 (57.5%)	127 (44.3%)	112 (86.8%)	<0.001
Mechanical ventilation, *n* (%)	173 (41.6%)	89 (31.0%)	84 (65.1%)	<0.001
HFNO, *n* (%)	116 (27.9%)	98 (34.1%)	18 (14.0%)	<0.001
Renal replacement therapy, *n* (%)	51 (12.3%)	21 (7.3%)	30 (23.3%)	<0.001
Comorbidities				
Hypertension, *n* (%)	195 (46.9%)	129 (44.9%)	66 (51.2%)	0.285
Diabetes mellitus, *n* (%)	135 (32.5%)	85 (29.6%)	50 (38.8%)	0.084
Heart failure (HF), *n* (%)	108 (26.0%)	59 (20.6%)	49 (38.0%)	<0.001
Coronary artery disease, *n* (%)	101 (24.3%)	56 (19.5%)	45 (34.9%)	0.001
CKD, *n* (%)	37 (8.9%)	18 (6.3%)	19 (14.7%)	0.005
Malignancy, *n* (%)	51 (12.3%)	32 (11.1%)	19 (14.7%)	0.385
Treatments				
Hydroxychloroquine, *n* (%)	76 (18.3%)	42 (14.6%)	34 (26.4%)	0.006
Plasmapheresis, *n* (%)	86 (20.7%)	62 (21.6%)	24 (18.6%)	0.570
Cytokine adsorption, *n* (%)	38 (9.1%)	22 (7.7%)	16 (12.4%)	0.172
Cultures obtained, *n* (%)	121 (29.1%)	81 (28.2%)	40 (31.0%)	0.644

Chi-squared or Fisher’s exact test for categorical variables; Mann–Whitney U test for age. HFNO, high-flow nasal oxygen; ICU, intensive care unit; CKD, chronic kidney disease.

**Table 2:** Mean ± SD of laboratory parameters (*n* = 416 unless stated).

Parameter	*n*	Mean ± SD
Procalcitonin (μg/L)	416	2.4 ± 11.34
Day 5 procalcitonin (μg/L)	336	3.6 ± 13.4
Day 5 lymphocytes (/μL)	336	781.68 ± 586.38
Day 5 ferritin (ng/mL)	336	937.42 ± 737.09
PaO_2_/FiO_2_ ratio (mmHg)	416	121.62 ± 32.36
Haemoglobin (g/dL)	416	12.41 ± 2.13
LDH (U/L)	416	512.49 ± 269.91
WBC (/μL)	416	9832.34 ± 4814.84
Day 5 WBC (/μL)	336	13,437.69 ± 14,115.09
Day 5 CRP (mg/dL)	336	9.57 ± 8.47

LDH, lactate dehydrogenase; WBC, white blood cell count; CRP, C-reactive protein; PaO_2_/FiO_2_, partial oxygen pressure/inspiratory oxygen fraction.

**Table 3:** Comparison of laboratory parameters by SOFA-2 score group.

Parameter	SOFA-2 0–4 Mean ± SD	SOFA-2 ≥ 5 Mean ± SD	*p*-Value
Procalcitonin (μg/L)	1.67 ± 8.84	4.03 ± 15.45	<0.001
Day 5 procalcitonin (μg/L)	2.23 ± 9.31	6.44 ± 19.06	<0.001
Day 5 lymphocytes (/μL)	824.64 ± 571.27	692.21 ± 609.66	0.009
Day 5 ferritin (ng/mL)	854.39 ± 682.35	1110.36 ± 816.25	0.001
PaO_2_/FiO_2_ ratio (mmHg)	126.35 ± 34.57	111.11 ± 23.76	<0.001
Haemoglobin (g/dL)	12.58 ± 2.04	12.03 ± 2.28	0.009
LDH (U/L)	488.08 ± 239.72	566.80 ± 321.71	0.010
WBC (/μL)	9433.41 ± 4244.61	10,719.88 ± 5809.97	0.056
Day 5 WBC (/μL)	13,068.06 ± 15,445.32	14,368.07 ± 10,967.48	0.008
Day 5 CRP (mg/dL)	8.11 ± 7.67	12.62 ± 9.25	<0.001

All comparisons: Mann–Whitney U test. LDH, lactate dehydrogenase; WBC, white blood cell count; CRP, C-reactive protein; PaO_2_/FiO_2_, partial oxygen pressure/inspiratory oxygen fraction.

**Table 4:** Comorbidities and hydroxychloroquine therapy by SOFA-2 score group.

Variable	SOFA-2 0–4 *n* (%)	SOFA-2 ≥ 5 *n* (%)	*p*-Value
Heart failure (HF)			<0.001
No	228 (79.4)	80 (62.0)	
Yes	59 (20.6)	49 (38.0)	
CKD			0.005
No	269 (93.7)	110 (85.3)	
Yes	18 (6.3)	19 (14.7)	
ASCVD			0.001
No	231 (80.5)	84 (65.1)	
Yes	56 (19.5)	45 (34.9)	
Hydroxychloroquine			0.004
No	245 (85.4)	95 (73.6)	
Yes	42 (14.6)	34 (26.4)	

Chi-squared test. CKD, chronic kidney disease; ASCVD, atherosclerotic cardiovascular disease.

**Table 5:** Multivariate logistic regression (stepwise model): associations of day 5 PCT and day 5 ferritin with SOFA-2 score.

Variable	OR	95% CI LL	95% CI UL	*p*-Value
Model A—Day 5 PCT (μg/L): Step 1 = covariates; Step 2 adds Day 5 PCT				
Step 1—significant covariates				
PaO_2_/FiO_2_ ratio (mmHg)	0.984	0.972	0.996	0.008
Haemoglobin (g/dL)	0.874	0.768	0.995	0.042
LDH (U/L)	1.001	1.000	1.002	0.006
Day 5 CRP (mg/dL)	1.049	1.015	1.084	0.004
ASCVD	2.509	1.011	6.225	0.047
Hydroxychloroquine	2.215	1.153	4.256	0.017
Step 2—Day 5 PCT added				
Day 5 PCT (μg/L)	1.001	0.981	1.022	0.887 (ns)
Model B—Day 5 ferritin (ng/mL): Step 1 = covariates; Step 2 adds Day 5 ferritin				
Step 1—significant covariates				
PaO_2_/FiO_2_ ratio (mmHg)	0.982	0.969	0.994	0.004
Haemoglobin (g/dL)	0.876	0.770	0.998	0.046
LDH (U/L)	1.001	1.000	1.002	0.005
Day 5 CRP (mg/dL)	1.052	1.019	1.086	0.002
Hydroxychloroquine	2.287	1.185	4.413	0.014
Step 2—Day 5 ferritin added				
Day 5 ferritin (ng/mL)	1.000	1.000	1.001	0.014 *

OR, odds ratio; CI, confidence interval; LL, lower limit; UL, upper limit; ns, not significant. Non-significant covariates omitted for clarity. * *p* = 0.014 after full adjustment including CRP, LDH, PaO_2_/FiO_2_, haemoglobin, comorbidities, and treatment. LDH, lactate dehydrogenase; CRP, C-reactive protein; ASCVD, atherosclerotic cardiovascular disease.

**Table 6:** Diagnostic performance of day 5 ferritin in predicting SOFA-2 score groups.

Parameter	Cut-Off (ng/mL)	AUC	Sensitivity % (95% CI)	Specificity % (95% CI)	+LR (95% CI)	−LR (95% CI)	+PV % (95% CI)	−PV % (95% CI)
Day 5 ferritin	>1191	0.608	35.78 (26.8–45.5)	82.38 (76.8–87.1)	2.03 (1.4–3.0)	0.78 (0.7–0.9)	49.4 (37.9–60.9)	72.8 (66.9–78.1)

+PV, positive predictive value; −PV, negative predictive value; +LR, positive likelihood ratio; −LR, negative likelihood ratio; CI, confidence interval; AUC, area under the curve.

**Table 7:** Laboratory parameters by day 5 PCT group [median (IQR)].

Parameter	PCT < 2 μg/L	PCT 2.01–10.00 μg/L	PCT ≥ 10.01 μg/L	*p*-Value
Procalcitonin (μg/L)	0.4 (0.2–0.7)	4.6 (3.0–7.2)	18.4 (11.4–33.8)	<0.001
Day 5 lymphocytes (/μL)	752 (380–1108)	582 (396–812)	488 (228–836)	0.027
Day 5 ferritin (ng/mL)	724 (320–1256)	1094 (658–1584)	1102 (704–1568)	0.001
PaO_2_/FiO_2_ ratio (mmHg)	114 (96–138)	112 (88–148)	106 (92–128)	0.636
Haemoglobin (g/dL)	12.5 (11.0–13.8)	12.2 (10.8–13.8)	12.3 (11.2–13.4)	0.938
LDH (U/L)	452 (320–628)	544 (390–748)	558 (382–848)	0.052
WBC (/μL)	9220 (6170–12,480)	9440 (6520–13,020)	9890 (6040–14,140)	0.987
Day 5 WBC (/μL)	10,520 (7280–14,680)	13,960 (10,060–19,260)	14,360 (9980–19,160)	<0.001
Day 5 CRP (mg/dL)	6.4 (2.8–11.4)	15.2 (9.6–22.8)	14.6 (8.4–24.2)	<0.001

Kruskal–Wallis test; results as median (IQR). LDH, lactate dehydrogenase; WBC, white blood cell count; CRP, C-reactive protein; PaO_2_/FiO_2_, partial oxygen pressure/inspiratory oxygen fraction.

**Table 8:** Comorbidities and treatment by day 5 PCT group.

Variable	PCT < 2 μg/L *n* (%)	PCT 2.01–10 μg/L *n* (%)	PCT ≥ 10.01 μg/L *n* (%)	*p*-Value
Heart failure (HF)				0.426
No	203 (74.4)	32 (76.2)	13 (61.9)	
Yes	70 (25.6)	10 (23.8)	8 (38.1)	
CKD				— ᵃ
No	256 (93.8)	38 (90.5)	15 (71.4)	
Yes	17 (6.2)	4 (9.5)	6 (28.6)	
ASCVD				0.572
No	209 (76.6)	31 (73.8)	14 (66.7)	
Yes	64 (23.4)	11 (26.2)	7 (33.3)	
Hydroxychloroquine				— ᵃ
No	218 (79.9)	32 (76.2)	17 (81.0)	
Yes	55 (20.1)	10 (23.8)	4 (19.0)	

ᵃ Chi-squared assumptions not met; *p*-values not calculated. CKD, chronic kidney disease; ASCVD, atherosclerotic cardiovascular disease.

**Table 9:** Laboratory parameters by day 5 ferritin group [median (IQR)].

Parameter	Ferritin 0–499 ng/mL	Ferritin 500–1000 ng/mL	Ferritin ≥ 1001 ng/mL	*p*-Value
Procalcitonin (μg/L)	0.4 (0.1–0.9)	0.5 (0.2–1.4)	1.0 (0.3–3.6)	<0.001
Day 5 procalcitonin (μg/L)	0.5 (0.2–1.1)	1.1 (0.4–3.3)	1.8 (0.6–5.2)	<0.001
Day 5 lymphocytes (/μL)	756 (374–1166)	724 (422–1076)	622 (290–1028)	0.118
PaO_2_/FiO_2_ ratio (mmHg)	116 (96–142)	112 (90–140)	108 (88–136)	0.359
Haemoglobin (g/dL)	11.3 (9.8–13.0)	12.7 (11.4–14.2)	12.4 (10.8–14.0)	<0.001
LDH (U/L)	398 (280–560)	442 (316–612)	578 (388–836)	<0.001
WBC (/μL)	9260 (5820–12,620)	8780 (5740–12,280)	9940 (6600–13,740)	0.201
Day 5 WBC (/μL)	9860 (6880–13,200)	11,420 (7360–15,380)	14,280 (8660–20,480)	<0.001
Day 5 CRP (mg/dL)	5.6 (2.0–10.6)	6.8 (2.6–13.0)	10.0 (4.4–18.6)	<0.001

Kruskal–Wallis test; results as median (IQR). LDH, lactate dehydrogenase; WBC, white blood cell count; CRP, C-reactive protein; PaO_2_/FiO_2_, partial oxygen pressure/inspiratory oxygen fraction.

**Table 10:** Comorbidities and treatment by day 5 ferritin group.

Variable	Ferritin 0–499 ng/mL *n* (%)	Ferritin 500–1000 ng/mL *n* (%)	Ferritin ≥ 1001 ng/mL *n* (%)	*p*-Value
Heart failure (HF)				0.772
No	71 (75.5)	97 (74.6)	80 (71.4)	
Yes	23 (24.5)	33 (25.4)	32 (28.6)	
CKD				0.359
No	89 (94.7)	120 (92.3)	100 (89.3)	
Yes	5 (5.3)	10 (7.7)	12 (10.7)	
ASCVD				0.837
No	73 (77.7)	98 (75.4)	83 (74.1)	
Yes	21 (22.3)	32 (24.6)	29 (25.9)	
Hydroxychloroquine				0.122
No	79 (84.0)	96 (73.8)	92 (82.1)	
Yes	15 (16.0)	34 (26.2)	20 (17.9)	

Chi-squared analysis. CKD, chronic kidney disease; ASCVD, atherosclerotic cardiovascular disease.

## Data Availability

The data supporting the findings of this study are available from the corresponding author upon reasonable request.

## Abbreviations

The following abbreviations are used in this manuscript:

Abbreviation	Definition
AOSD	Adult-Onset Still’s Disease
ARDS	Acute Respiratory Distress Syndrome
ASCVD	Atherosclerotic Cardiovascular Disease
AUC	Area Under the Curve
BAL	Bronchoalveolar Lavage
CI	Confidence Interval
CKD	Chronic Kidney Disease
COVID-19	Coronavirus Disease 2019
CRP	C-Reactive Protein
eGFR	Estimated Glomerular Filtration Rate
HF	Heart Failure
HFNO	High-Flow Nasal Oxygen
HLH	Haemophagocytic Lymphohistiocytosis
ICU	Intensive Care Unit
IFN	Interferon
IL	Interleukin
IQR	Interquartile Range
LDH	Lactate Dehydrogenase
MAS	Macrophage Activation Syndrome
OR	Odds Ratio
PCT	Procalcitonin
ROC	Receiver Operating Characteristic
SARS-CoV-2	Severe Acute Respiratory Syndrome Coronavirus 2
SD	Standard Deviation
SOFA	Sepsis-related Organ Failure Assessment
TLR	Toll-Like Receptor
TNF-α	Tumour Necrosis Factor-Alpha
WBC	White Blood Cell Count
WHO	World Health Organization
